# The genome sequence of a barkfly, *Mesopsocus fuscifrons *Meinander, 1966

**DOI:** 10.12688/wellcomeopenres.20641.1

**Published:** 2024-02-19

**Authors:** Duncan Sivell, Natural History Museum Genome Acquisition Lab, Darwin Tree of Life Barcoding collective, Wellcome Sanger Institute Tree of Life Management, Samples and Laboratory team, Wellcome Sanger Institute Scientific Operations: Sequencing Operations, Wellcome Sanger Institute Tree of Life Core Informatics team, Tree of Life Core Informatics collective, Darwin Tree of Life Consortium

**Affiliations:** 1 Natural History Museum, London, England, UK

**Keywords:** Mesopsocus fuscifrons, a barkfly, genome sequence, chromosomal, Psocodea

## Abstract

We present a genome assembly from an individual female *Mesopsocus fuscifrons* (barkfly; Arthropoda; Insecta; Psocodea; Mesopsocidae). The genome sequence is 184.3 megabases in span. Most of the assembly is scaffolded into 9 chromosomal pseudomolecules. The mitochondrial genome has also been assembled and is 20.13 kilobases in length.

## Species taxonomy

Eukaryota; Metazoa; Eumetazoa; Bilateria; Protostomia; Ecdysozoa; Panarthropoda; Arthropoda; Mandibulata; Pancrustacea; Hexapoda; Insecta; Dicondylia; Pterygota; Neoptera; Paraneoptera; Psocodea; Psocoptera; Psocomorpha; Homilopsocidea; Peripsocoidea; Mesopsocidae; *Mesopsocus*; *Mesopsocus fuscifrons* (Meinander, 1966) (NCBI:txid2866285).

## Background

*Mesopsocus fuscifrons* is a large, 3.5–5.0 mm long, barkfly (Psocodea: ‘Psocoptera’) that can be recognised by its very dark frons and distinctive eye pattern ( Lienhard, 1998). The wings are fully formed in males, but reduced to vestigial wing buds in females. Female *M. fuscifrons* also have dark rings near the base of the tibiae that are characteristic of this species. The eye pattern can be described as a dark chevron that lies horizontally across the eye. In practice the eyes appear dark with a paler grey line running horizontally across their middle. Either interpretation is clearly different from the eyes of other British *Mesopsocus*, which can be identified using New (2005).

*Mesopsocus fuscifrons* is a recent addition to the British fauna with a population in the Wildlife Garden of the Natural History Museum in South Kensington ( McCarter *et al.*, 2021). Specimens captured in a Malaise trap in the summer of 2013 are the earliest British records we are aware of. This species has also been found at three sites in Richmond and Ham ( Cook, 2023), and undoubtedly occurs elsewhere in the Greater London area.

This barkfly is a Mediterranean species known from Morocco, Algeria, France, Italy, Macedonia and Greece ( Lienhard, 1998) that has expanded its range into northern Europe in the past decade. *Mesopsocus fuscifrons* is now recorded in Germany, northern France, Belgium, the Netherlands and Denmark as well as the UK ( Schuch *et al.*, 2021). There were also records from southern Sweden in 2006 but it is not clear if a population persists there ( Svensson & Hall, 2010).

*Mesopsocus fuscifrons* has been found on a wide variety of broadleaved trees and shrubs that may or may not have lichens growing on the bark ( Cook, 2023; Schuch *et al.*, 2021). Lienhard (1998) also reports *M. fuscifrons* living on conifers. Across its geographic range *M. fuscifrons* adults may be found in any month of the year, though are more likely to be seen from May to October ( Schuch *et al.*, 2021). British records so far have been made from May to August ( Cook, 2023).

A single female observed on the trunk of a young ash tree ( *Fraxinus excelsior*) in the NHM Wildlife Garden was captured and submitted to the Darwin Tree of Life project on 8 June 2021. It has been suggested that *Mesopsocus fuscifrons* may be synonymous with *M. ypsilon* Ball, 1937, a species known from Morocco and Greece ( Lienhard, 1998). The publication of this genome sequence will help to answer this question.

## Genome sequence report

**Figure 1.**
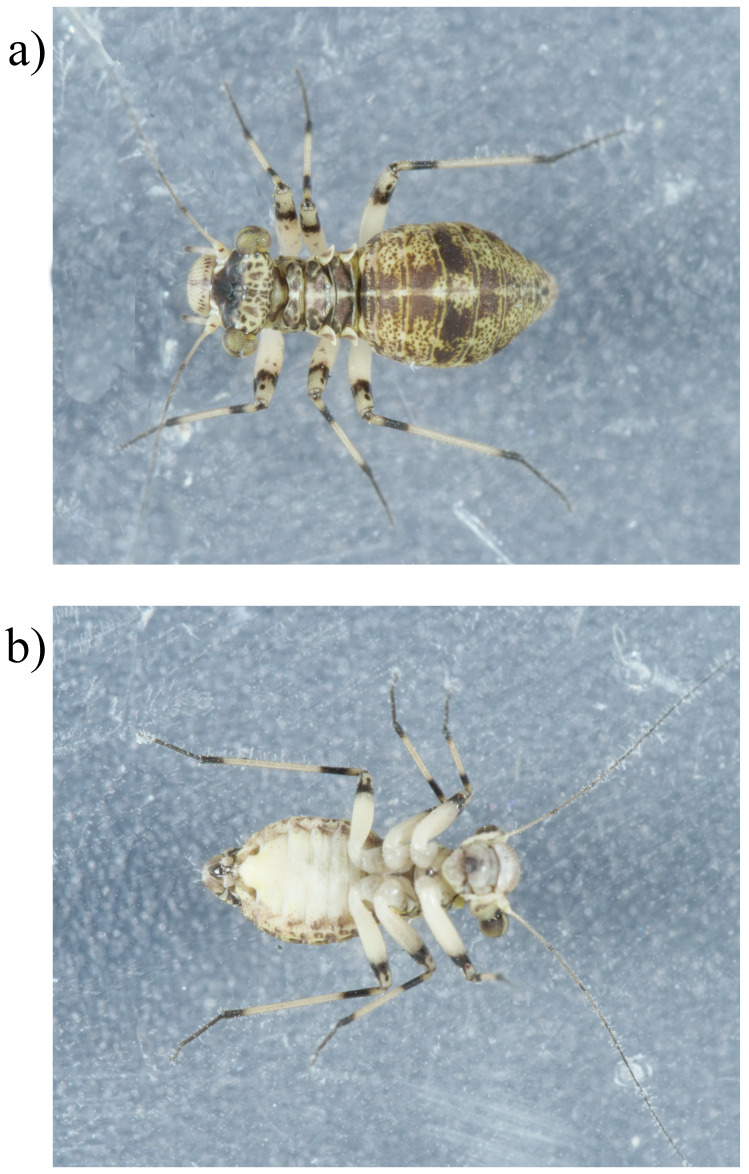
Photographs of the *Mesopsocus fuscifrons* (iuMesFusc1, NHMUK014043153) specimen used for genome sequencing **a**) dorsal view, **b**) ventral view.

The genome was sequenced from one female *Mesopsocus fuscifrons* (NHMUK014043153, Figure 1) collected from Natural History Museum Wildlife Garden, England (51.5, –0.18). A total of 93-fold coverage in Pacific Biosciences single-molecule HiFi long reads was generated. Primary assembly contigs were scaffolded with chromosome conformation Hi-C data. Manual assembly curation corrected 204 missing joins or mis-joins and removed 106 haplotypic duplications, reducing the assembly length by 4.60% and the scaffold number by 75.29%, and increasing the scaffold N50 by 13.37%.

**Figure 2.**
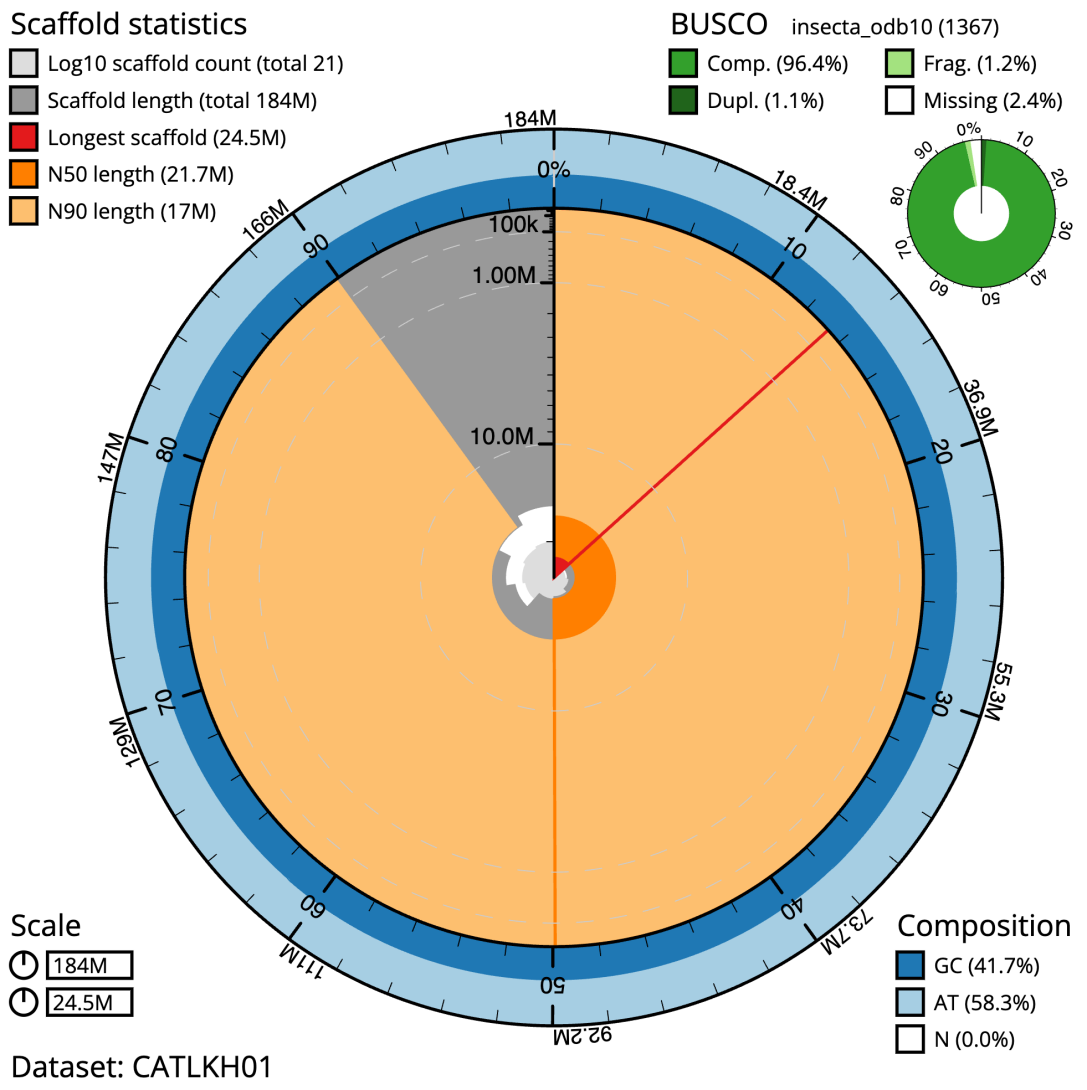
Genome assembly of *Mesopsocus fuscifrons*, iuMesFusc1.1: metrics. The BlobToolKit Snailplot shows N50 metrics and BUSCO gene completeness. The main plot is divided into 1,000 size-ordered bins around the circumference with each bin representing 0.1% of the 184,352,988 bp assembly. The distribution of scaffold lengths is shown in dark grey with the plot radius scaled to the longest scaffold present in the assembly (24,526,141 bp, shown in red). Orange and pale-orange arcs show the N50 and N90 scaffold lengths (21,655,844 and 16,975,629 bp), respectively. The pale grey spiral shows the cumulative scaffold count on a log scale with white scale lines showing successive orders of magnitude. The blue and pale-blue area around the outside of the plot shows the distribution of GC, AT and N percentages in the same bins as the inner plot. A summary of complete, fragmented, duplicated and missing BUSCO genes in the insecta_odb10 set is shown in the top right. An interactive version of this figure is available at https://blobtoolkit.genomehubs.org/view/CATLKH01/dataset/CATLKH01/snail.

**Figure 3.**
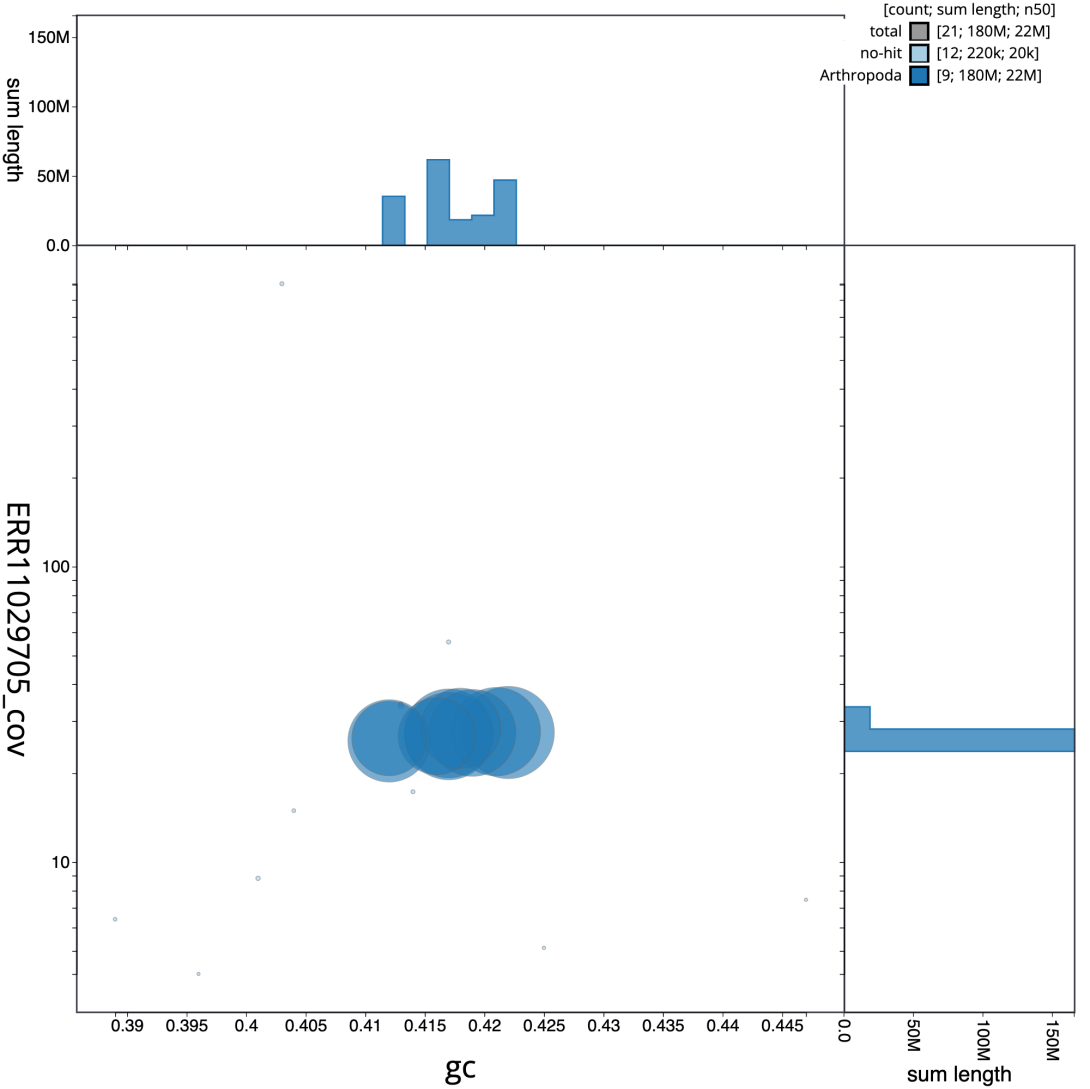
Genome assembly of *Mesopsocus fuscifrons*, iuMesFusc1.1: BlobToolKit GC-coverage plot. Scaffolds are coloured by phylum. Circles are sized in proportion to scaffold length. Histograms show the distribution of scaffold length sum along each axis. An interactive version of this figure is available at https://blobtoolkit.genomehubs.org/view/CATLKH01/dataset/CATLKH01/blob.

**Figure 4.**
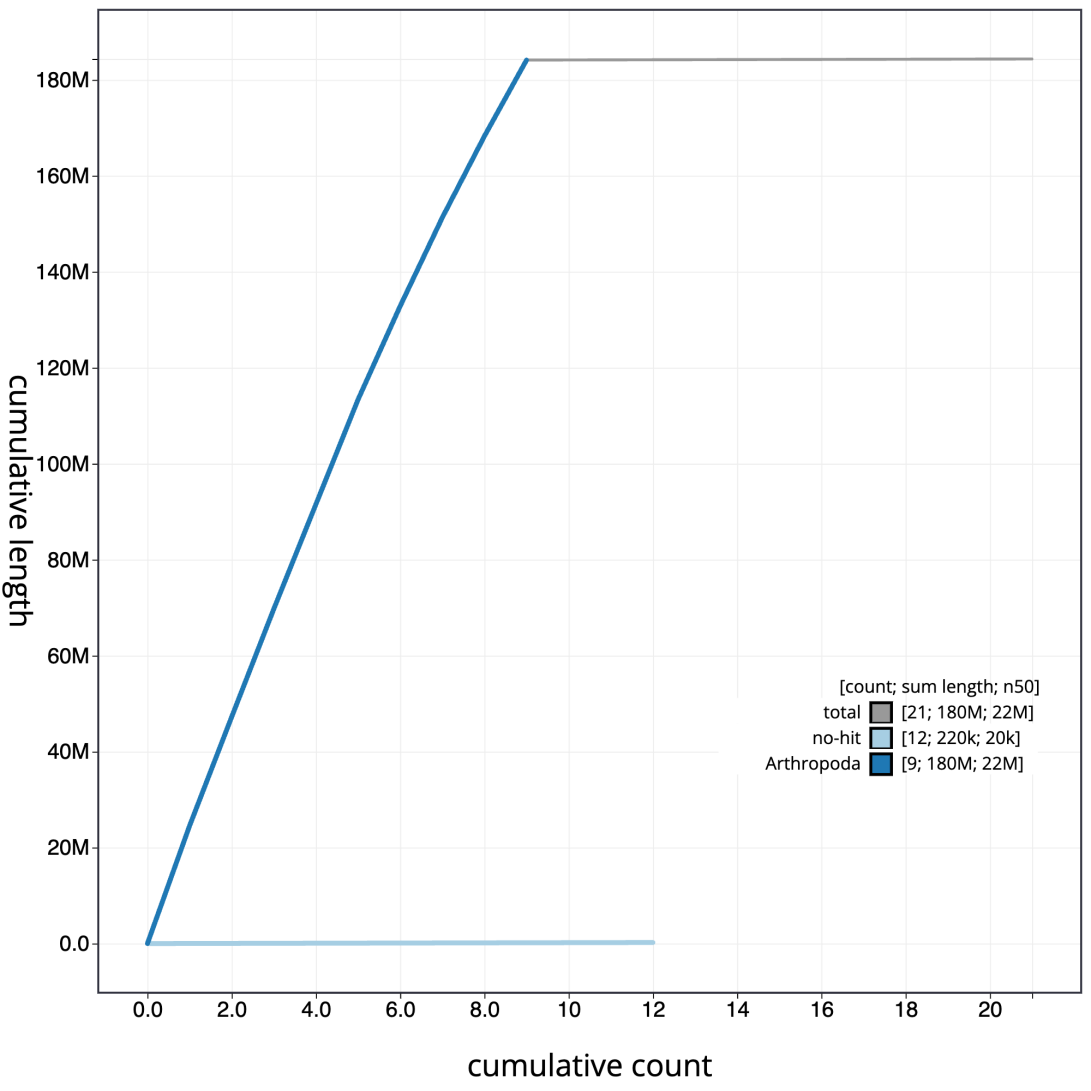
Genome assembly of *Mesopsocus fuscifrons*, iuMesFusc1.1: BlobToolKit cumulative sequence plot. The grey line shows cumulative length for all scaffolds. Coloured lines show cumulative lengths of scaffolds assigned to each phylum using the buscogenes taxrule. An interactive version of this figure is available at https://blobtoolkit.genomehubs.org/view/CATLKH01/dataset/CATLKH01/cumulative.

**Figure 5.**
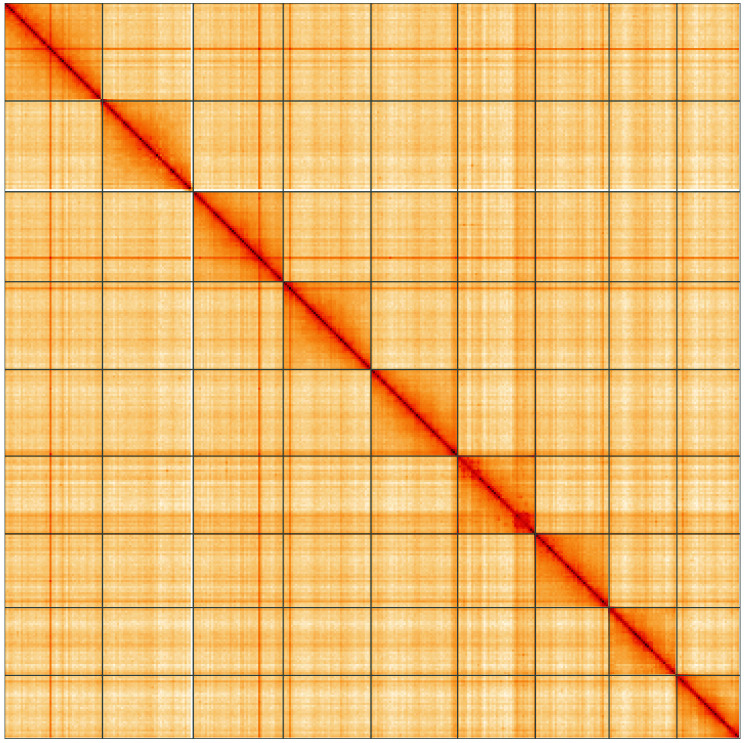
Genome assembly of *Mesopsocus fuscifrons*, iuMesFusc1.1: Hi-C contact map of the iuMesFusc1.1 assembly, visualised using HiGlass. Chromosomes are shown in order of size from left to right and top to bottom. An interactive version of this figure may be viewed at https://genome-note-higlass.tol.sanger.ac.uk/l/?d=I7ipiU4sRtSk_uJrqsXM3Q.

The final assembly has a total length of 184.3 Mb in 20 sequence scaffolds with a scaffold N50 of 21.7 Mb ( Table 1). The snailplot in Figure 2 provides a summary of the assembly statistics, while the distribution of assembly scaffolds on GC proportion and coverage is shown in Figure 3. The cumulative assembly plot in Figure 4 shows curves for subsets of scaffolds assigned to different phyla. Most (99.9%) of the assembly sequence was assigned to 9 chromosomal-level scaffolds, representing 9 autosomes. Chromosome-scale scaffolds confirmed by the Hi-C data are named in order of size ( Figure 5; Table 2). Sex chromosomes were not identified due to the lack of comparators within this order. Psocodea have multiple sex determination mechanisms ( Hodson *et al.*, 2017). While not fully phased, the assembly deposited is of one haplotype. Contigs corresponding to the second haplotype have also been deposited. The mitochondrial genome was also assembled and can be found as a contig within the multifasta file of the genome submission.

**Table 1.** Genome data for *Mesopsocus fuscifrons*, iuMesFusc1.1.

Project accession data		
Assembly identifier	iuMesFusc1.1	
Species	*Mesopsocus fuscifrons*	
Specimen	iuMesFusc1	
NCBI taxonomy ID	2866285	
BioProject	PRJEB60722	
BioSample ID	SAMEA10241719	
Isolate information	iuMesFusc1, female: whole organism (DNA sequencing and Hi-C sequencing)	
Assembly metrics *		*Benchmark*
Consensus quality (QV)	58.2	*≥ 50*
*k*-mer completeness	100.0%	*≥ 95%*
BUSCO **	C:96.4%[S:95.3%,D:1.1%], F:1.2%,M:2.4%,n:1,367	*C ≥ 95%*
Percentage of assembly mapped to chromosomes	99.9%	*≥ 95%*
Sex chromosomes	Not identified	*localised homologous * *pairs*
Organelles	Mitochondrial genome: 20.13 kb	*complete single alleles*
Raw data accessions		
PacificBiosciences SEQUEL II	ERR11029705	
Hi-C Illumina	ERR11042967	
Genome assembly		
Assembly accession	GCA_950004255.1	
*Accession of alternate haplotype*	GCA_949987595.1	
Span (Mb)	184.3	
Number of contigs	276	
Contig N50 length (Mb)	1.2	
Number of scaffolds	20	
Scaffold N50 length (Mb)	21.7	
Longest scaffold (Mb)	24.53	

* Assembly metric benchmarks are adapted from column VGP-2020 of “Table 1: Proposed standards and metrics for defining genome assembly quality” from ( Rhie *et al.*, 2021). ** BUSCO scores based on the insecta_odb10 BUSCO set using version 5.3.2. C = complete [S = single copy, D = duplicated], F = fragmented, M = missing, n = number of orthologues in comparison. A full set of BUSCO scores is available at https://blobtoolkit.genomehubs.org/view/CATLKH01/dataset/CATLKH01/busco.

**Table 2.** Chromosomal pseudomolecules in the genome assembly of *Mesopsocus fuscifrons*, iuMesFusc1.

INSDC accession	Chromosome	Length (Mb)	GC%
OX465373.1	1	24.53	42.0
OX465374.1	2	22.71	41.5
OX465375.1	3	22.5	42.0
OX465376.1	4	21.98	41.5
OX465377.1	5	21.66	42.0
OX465378.1	6	19.48	41.0
OX465379.1	7	18.44	42.0
OX465380.1	8	16.98	41.5
OX465381.1	9	15.86	41.0
OX465382.1	MT	0.02	40.5

The estimated Quality Value (QV) of the final assembly is 58.2 with *k*-mer completeness of 100.0%, and the assembly has a BUSCO v5.3.2 completeness of 96.4% (single = 95.3%, duplicated = 1.1%), using the insecta_odb10 reference set ( *n* = 1,367).

Metadata for specimens, barcode results, spectra estimates, sequencing runs, contaminants and pre-curation assembly statistics are given at https://links.tol.sanger.ac.uk/species/2866285.

## Methods

### Sample acquisition and nucleic acid extraction

A female *Mesopsocus fuscifrons* (specimen ID NHMUK014043153, ToLID iuMesFusc1) was observed on the trunk of a young ash tree in the Natural History Museum Wildlife Garden, England, UK (latitude 51.5, longitude –0.18) on 2021-06-08. The specimen was collected and identified by Duncan Sivell (Natural History Museum) and then dry frozen at –80 °C.

The workflow for high molecular weight (HMW) DNA extraction at the Wellcome Sanger Institute (WSI) includes a sequence of core procedures: sample preparation; sample homogenisation, DNA extraction, fragmentation, and clean-up. The sample was prepared in the WSI Tree of Life Core laboratory: the iuMesFusc1 sample was weighed and dissected on dry ice ( Jay *et al.*, 2023). Tissue from the whole organism was homogenised using a PowerMasher II tissue disruptor ( Denton *et al.*, 2023a).

HMW DNA was extracted in the WSI Scientific Operations core using the Automated MagAttract v2 protocol ( Oatley *et al.*, 2023). HMW DNA was sheared into an average fragment size of 12–20 kb in a Megaruptor 3 system with speed setting 31 ( Bates *et al.*, 2023). Sheared DNA was purified by solid-phase reversible immobilisation ( Strickland *et al.*, 2023): in brief, the method employs a 1.8X ratio of AMPure PB beads to sample to eliminate shorter fragments and concentrate the DNA. The concentration of the sheared and purified DNA was assessed using a Nanodrop spectrophotometer and Qubit Fluorometer and Qubit dsDNA High Sensitivity Assay kit. Fragment size distribution was evaluated by running the sample on the FemtoPulse system.

Protocols developed by the Wellcome Sanger Institute (WSI) Tree of Life core laboratory are publicly available on protocols.io ( Denton *et al.*, 2023b).

### Sequencing

Pacific Biosciences HiFi circular consensus DNA sequencing libraries were constructed according to the manufacturers’ instructions. DNA sequencing was performed by the Scientific Operations core at the WSI on a Pacific Biosciences SEQUEL II (HiFi) instrument. Hi-C data were also generated from remaining tissue of iuMesFusc1 using the Arima2 kit and sequenced on the Illumina NovaSeq 6000 instrument.

### Genome assembly, curation and evaluation

Assembly was carried out with Hifiasm ( Cheng *et al.*, 2021) and haplotypic duplication was identified and removed with purge_dups ( Guan *et al.*, 2020). The assembly was then scaffolded with Hi-C data ( Rao *et al.*, 2014) using YaHS ( Zhou *et al.*, 2023). The assembly was checked for contamination and corrected using the gEVAL system ( Chow *et al.*, 2016) as described previously ( Howe *et al.*, 2021). Manual curation was performed using gEVAL, HiGlass ( Kerpedjiev *et al.*, 2018) and Pretext ( Harry, 2022). The mitochondrial genome was assembled using MitoHiFi ( Uliano-Silva *et al.*, 2023), which runs MitoFinder ( Allio *et al.*, 2020) or MITOS ( Bernt *et al.*, 2013) and uses these annotations to select the final mitochondrial contig and to ensure the general quality of the sequence.

A Hi-C map for the final assembly was produced using bwa-mem2 ( Vasimuddin *et al.*, 2019) in the Cooler file format ( Abdennur & Mirny, 2020). To assess the assembly metrics, the *k*-mer completeness and QV consensus quality values were calculated in Merqury ( Rhie *et al.*, 2020). This work was done using Nextflow ( Di Tommaso *et al.*, 2017) DSL2 pipelines “sanger-tol/readmapping” ( Surana *et al.*, 2023a) and “sanger-tol/genomenote” ( Surana *et al.*, 2023b). The genome was analysed within the BlobToolKit environment ( Challis *et al.*, 2020) and BUSCO scores ( Manni *et al.*, 2021; Simão *et al.*, 2015) were calculated.

Table 3 contains a list of relevant software tool versions and sources.

**Table 3.** Software tools: versions and sources.

Software tool	Version	Source
BlobToolKit	4.1.7	https://github.com/blobtoolkit/blobtoolkit
BUSCO	5.3.2	https://gitlab.com/ezlab/busco
Hifiasm	0.16.1-r375	https://github.com/chhylp123/hifiasm
HiGlass	1.11.6	https://github.com/higlass/higlass
Merqury	MerquryFK	https://github.com/thegenemyers/MERQURY.FK
MitoHiFi	3	https://github.com/marcelauliano/MitoHiFi
PretextView	0.2	https://github.com/wtsi-hpag/PretextView
purge_dups	1.2.5	https://github.com/dfguan/purge_dups
sanger-tol/genomenote	v1.0	https://github.com/sanger-tol/genomenote
sanger-tol/readmapping	1.1.0	https://github.com/sanger-tol/readmapping/tree/1.1.0
YaHS	1.2a.2	https://github.com/c-zhou/yahs

### Wellcome Sanger Institute – Legal and Governance

The materials that have contributed to this genome note have been supplied by a Darwin Tree of Life Partner. The submission of materials by a Darwin Tree of Life Partner is subject to the **‘Darwin Tree of Life Project Sampling Code of Practice’**, which can be found in full on the Darwin Tree of Life website here. By agreeing with and signing up to the Sampling Code of Practice, the Darwin Tree of Life Partner agrees they will meet the legal and ethical requirements and standards set out within this document in respect of all samples acquired for, and supplied to, the Darwin Tree of Life Project. 

Further, the Wellcome Sanger Institute employs a process whereby due diligence is carried out proportionate to the nature of the materials themselves, and the circumstances under which they have been/are to be collected and provided for use. The purpose of this is to address and mitigate any potential legal and/or ethical implications of receipt and use of the materials as part of the research project, and to ensure that in doing so we align with best practice wherever possible. The overarching areas of consideration are: 

•   Ethical review of provenance and sourcing of the material 

•   Legality of collection, transfer and use (national and international) 

Each transfer of samples is further undertaken according to a Research Collaboration Agreement or Material Transfer Agreement entered into by the Darwin Tree of Life Partner, Genome Research Limited (operating as the Wellcome Sanger Institute), and in some circumstances other Darwin Tree of Life collaborators.

## Data Availability

European Nucleotide Archive: *Mesopsocus fuscifrons*. Accession number PRJEB60722; https://identifiers.org/ena.embl/PRJEB60722 ( Wellcome Sanger Institute, 2023). The genome sequence is released openly for reuse. The *Mesopsocus fuscifrons* genome sequencing initiative is part of the Darwin Tree of Life (DToL) project. All raw sequence data and the assembly have been deposited in INSDC databases. The genome will be annotated using available RNA-Seq data and presented through the Ensembl pipeline at the European Bioinformatics Institute. Raw data and assembly accession identifiers are reported in Table 1.

## Author information

Members of the Natural History Museum Genome Acquisition Lab are listed here: https://doi.org/10.5281/zenodo.7139035.

Members of the Darwin Tree of Life Barcoding collective are listed here: https://doi.org/10.5281/zenodo.4893703.

Members of the Wellcome Sanger Institute Tree of Life Management, Samples and Laboratory team are listed here: https://doi.org/10.5281/zenodo.10066175.

Members of Wellcome Sanger Institute Scientific Operations: Sequencing Operations are listed here: https://doi.org/10.5281/zenodo.10043364.

Members of the Wellcome Sanger Institute Tree of Life Core Informatics team are listed here: https://doi.org/10.5281/zenodo.10066637.

Members of the Tree of Life Core Informatics collective are listed here: https://doi.org/10.5281/zenodo.5013541.

Members of the Darwin Tree of Life Consortium are listed here: https://doi.org/10.5281/zenodo.4783558.
