# Supplementary material for: The genome sequence of a barkfly, Mesopsocus fuscifrons Meinander, 1966
Source: Wellcome Open Res. 2024 Feb 19;9:72. [Version 1] doi: 10.12688/wellcomeopenres.20641.1 (PMC11303940; doi:10.12688/wellcomeopenres.20641.1)
Supplement: Peer review history

# Reviewer report: Reviewer response for version 1

**DOI:** 10.21956/wellcomeopenres.22844.r90223

**Published:** 2024-08-06

**Authors:** Jae Hak Son

**Affiliations:** 1 Rutgers, The State University of New Jersey, Piscataway, USA

This study reports a high-quality genome assembly of barkfly at the chromosome level.

  The rationale for why this study is reported and the dataset is created is clearly described.

 The protocol described here is reasonable. However, if some details in sections of the genome assembly and the sample acquisition and DNA extraction are provided, the article would be improved. This is a minor comment that does not affect the approval. See the below for the detailed comments.

 It is mentioned that the remaining tissue was used for HiC data after PacBio Hifi sequencing. Here, it is not described which tissues were used for PacBio Hifi and HiC sequencing. For genome assembly, it would be better to show which parameters are exactly used in each software program. 

 Can the Hi-C contact map show the contigs assembled into scaffolds in the figure? I think that this would help to assess how the initial assembly (contig-level) contribute to the scaffolded assembly.

Are sufficient details of methods and materials provided to allow replication by others?

Partly

Is the rationale for creating the dataset(s) clearly described?

Yes

Are the datasets clearly presented in a useable and accessible format?

Yes

Are the protocols appropriate and is the work technically sound?

Yes

Reviewer Expertise:

Evolutionary genomics

I confirm that I have read this submission and believe that I have an appropriate level of expertise to confirm that it is of an acceptable scientific standard.

# Reviewer report: Reviewer response for version 1

**DOI:** 10.21956/wellcomeopenres.22844.r77856

**Published:** 2024-04-22

**Authors:** Kosuke Kataoka

**Affiliations:** 1 Waseda University, Tokyo, Japan

The author present a genome assembly of *Mesopsocus fuscifrons* (Insecta; Psocodea) using PacBio HiFi and Arima-based chromosome proximity analysis. The genome assembly display high degree continuity and is examined with attention to mis-assembly and contamination. The manuscript is well-written, however, there are several concerns and questions:

 1. The total size of genome assembly is matching genome size determined by experimental methods like flow cytometry? If there is no data on genome size estimation, the genome size of the assembly is close to those of their relative species?

 2. The author present 9 chromosomal-level scaffolds. Are the numbers close and reliable to those estimated from the number of chromosomes in closely related species? 

 3. Is the DNA for the sequencing derived from the whole body?

Are sufficient details of methods and materials provided to allow replication by others?

Yes

Is the rationale for creating the dataset(s) clearly described?

Yes

Are the datasets clearly presented in a useable and accessible format?

Yes

Are the protocols appropriate and is the work technically sound?

Yes

Reviewer Expertise:

Genomics, Omics analysis

I confirm that I have read this submission and believe that I have an appropriate level of expertise to confirm that it is of an acceptable scientific standard.
